# Prevalence of First Permanent Molar Caries in and Its Relationship to the Dental Knowledge of 9–12-Year Olds from Jeddah, Kingdom of Saudi Arabia

**DOI:** 10.5402/2012/391068

**Published:** 2012-03-11

**Authors:** Khalid H. M. Al-Samadani, Mohammad Sami Ahmad

**Affiliations:** ^1^Department of Restorative Dental Sciences, College of Dentistry, Taibah University, P.O. Box 2898, Madinah Al Munawwarah 43353, Saudi Arabia; ^2^Department of Preventive Dental Sciences, College of Dentistry, Taibah University, P.O. Box 2898, Madinah Al Munawwarah 43353, Saudi Arabia

## Abstract

The carious status of the first permanent molar (FPM) was studied in 432 school children (aged 9–12 years) from a randomly selected primary schools from Sharfia area of Jeddah, Kingdom of Saudi Arabia. The sample consisted of 108 children from each age group of 9, 10, 11, and 12 years old. In total, 24.5% had all of their FPMs sound and 6% had all FPMs carious. The prevalence of four sound FPMs varied according to age with the highest (33%) amongst the nine-year olds and the lowest (16.5%) in the oldest children (12 years). Almost one-third (32.5%) of the children, who knew the age of eruption of the FPMs, had all of their molars sound. The children who had received advice regarding oral hygiene from a dentist or parent had more sound FPMs compared to the children who did not receive any advice. The number of carious FPMs increased with age. The prevalence of caries of the FPM was high and increased with increasing age. The level of knowledge had a positive correlation with the caries levels amongst this cohort of scholars.

## 1. Introduction

Jeddah is a commercial city situated at the western coast of Saudi Arabia. In the Sharfia district of Jeddah, majority of people are from India, Pakistan, and other Asian countries. In this region, there have been no studies undertaken to determine the prevalence of dental caries in first permanent molars (FPMs). In recent years, the global distribution of dental caries presents a varied picture; most of the countries with low caries prevalence are experiencing an unprecedented increase in caries prevalence and severity of dental caries including Saudi Arabia. On the other hand, in several industrialized countries, a reduction of dental caries incidence and improvement of gingival health care are evident [1, 2]. This decline in dental caries is mainly due to appropriate use of fluorides and preventive oral health measures [3, 4]. Dental caries is one of the most highly prevalent diseases in children, and the FPMs are important as they are very prone to caries because of their anatomical structure and early eruption in the mouth. As a result, many children have to visit the dental professional requiring restorations or extractions of these molars. This is expensive, time consuming, and often traumatic for the young child. Hence the prevention of dental caries remains an important responsibility of the dental profession. Many studies [3, 4] have stressed the importance of oral hygiene instruction, the regular use of topical and systemic fluoride, and the application of fissure sealants in the prevention of dental caries especially on the first permanent molars. Several studies have reported high dental caries prevalence in school children from Saudi Arabia and other developing countries [5–11]. The frequency of involvement of dental surfaces by caries lesions varies with age, and peak of intensity occurs during certain stages of human life [12]. 

The incidence of caries among the various teeth varies considerably. The morphology, time of eruption, and positioning of the tooth in the oral cavity confer inherited disadvantages or advantages to the various methods used in the control of plaque and hence tooth decay and losses. A study conducted in Nigeria showed that the FPMs accounted for 42% of all extractions due to caries which is the highest when compared to other teeth [13]. In Taiwan 48% of children aged 6 years were caries free in their permanent first molars [14]. The caries status of the FPM was studied among 13–16-year-old school children from Sri Lanka, and it was found that, in 36% of cases, all four molars were sound while 11% had all four FPMs affected by caries [15]. A Japanese study showed that most of occlusal caries occurred 1-2 years after their eruption [16]. Caries studies in the Kingdom of Saudi Arabia showed prevalence from 68 to 87% among primary school children [17–20]. These studies showed that, with increasing age, there was an increase in the prevalence of dental caries of the first permanent molars.

In Jeddah, similar to other Saudi cities, the prevalence of dental caries is high and it is essential to obtain base line data regarding the condition of the first permanent molars so that appropriate prevention and treatment options can be implemented. This study was unique as it set out to determine the baseline data of a community that was neglected. This data will serve as a basis for the introduction and monitoring of intervention and educational oral health programmes for this community. Follow-up studies will be done on an annual basis to monitor the outcomes and if necessary modify and improve the oral health package that will be recommended. Depending on the results obtained, relevant and practical recommendations will be suggested.

The aim of the study was to determine the prevalence of dental caries in the first permanent molars among 9–12-year-old school children from the Sharfia district, Jeddah. The second aim was to correlate the prevalence of caries in relation with the dental knowledge.

## 2. Methods

**Figure 1:**
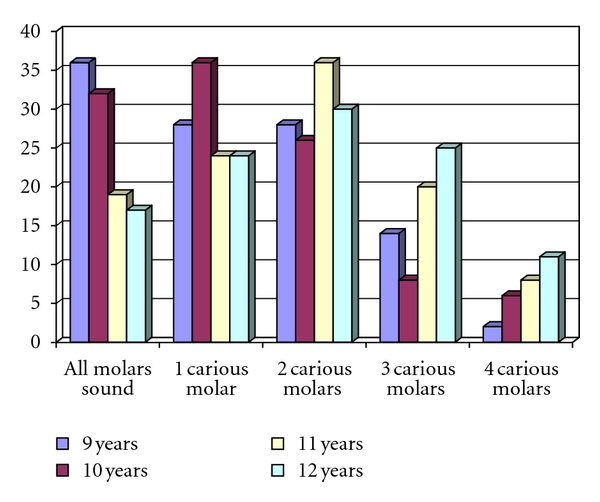


A cross-sectional analytical study design was used to determine the prevalence of dental caries in the FPMs. A total of 432 school children were examined from randomly selected primary school in the Sharfia district, Jeddah. The sample consisted of a total of 108 male and female children from each age group of 9, 10, 11, and 12 years (Figure 1). The sample size for the study was determined using pathfinder survey technique as described by the World Health Organization (WHO) [21]. Consent for examination of the children was obtained from the respective headmaster of the schools. The primary school children were examined by two trained and calibrated examiners. Kappa statistic 0.9 and 0.89 was observed for both examiners [22–24]. By the WHO standards, the acceptable consistency that an examiner should attempt to achieve is 0.80 [21]. 

The WHO criteria were utilized to diagnose the carious status of the first permanent molars [21]. Only first permanent molars of the children were recorded. Missing first permanent molars due to caries was recorded as “carious.” All first molars that were restored were classified as “carious” and those with fissure sealants were classified as “sound.” The examination was conducted on a simple sitting chair in normal day light with the help of wooden tongue depressor. In case of any doubt, the tooth was marked as sound.

The questionnaire was developed from other studies and was piloted amongst 20 school children who attended the Primary Health Clinic for dental treatment. The questionnaire consisted of four closed ended questions and elicited information regarding the knowledge of eruption of the first permanent molar, the frequency of visiting a dentist, and the advice he/she received from the dentist and from the parents about cleaning their teeth after consuming sugary foods. The questionnaire was in English, and the assistant interviewed the children after the examiner completed the dental examination. The assistant was unaware of the caries prevalence when administering the questionnaire and was hence blinded which reduced bias.

Statistical package for social science (Window version 15) was used to generate descriptive statistics and inferential tests. Appropriate statistical tests were used, and a *P* level less than 0.05 was considered significant.

The children who required dental treatment were referred to the Primary Health Clinic and Specialty hospitals. Those who had sound molars were also referred for fissure sealants and fluoride applications. All of the children received oral hygiene instructions and an oral hygiene hamper which consisted of toothpaste and a toothbrush.

## 3. Results

Out of the total of 432 children, 199 (46%) were male and 233 (54%) were female. As there was no significant difference between the genders in terms of caries status and the age of the children, the data was grouped together for ease of the statistical analysis. In total 106 (24.5%) had all FPMs sound. The remaining 326 (75.5%) children had one or more carious first permanent molars. A quarter of the children, 112 (26%), had one molar carious, 120 (28%) had two molars carious, 67 (15.5%) had three molars carious, and 27 (6%) had all four molars carious. The breakdown according to the age is shown in Table 1. The carious status of all FPMs increased significantly with an increase in age of the children (*P* < 0.01). The caries prevalence amongst all of the age groups was considerably high; in the 9-year-old category, two thirds (67%) had one or more carious permanent molar, and this number increased to over 80% in the 12-year-old children; 10% of them had all four of their FPMs decayed.

Less than 80 (20%) of the respondents chose the correct option regarding the eruption time of the FPM. Amongst them, 32.5% had all four FPMs sound and 5 (6.5%) had all four molars carious. Of those who did not choose the correct eruption age, 80 (22.5%) had all molars sound and 6.5% had all four molars carious (Table 2). The difference between the two groups regarding their caries status of the FPMs was not statistically significant (*P* > 0.05).

About half of the children, 222 (51%), visited the dentist in the last six months. Amongst them, 66 (29.5%) had all of their FPMs sound, and only 3% had all four molars carious. Of the remaining children, 19% had sound first molars and 9.5% had all four molars carious (Table 3). The difference between the two groups in their FPMS carious status was statistically significant (*P* < 0.05).

Of those children who visited the dentist (222), just over 27 (10%) got advice about cleaning their teeth after eating sugary food or drink. Amongst them 37.5% had all four FPMs sound (Table 4). From the remaining 88% who did not get any advice, almost a quarter, 45 (23%), had all permanent molars sound and 23 (6%) had all permanent molars decayed. The difference between the two groups in relation to their caries status was statistically significant (*P* < 0.05).

A total of 256 (59.5%) children received advice about cleaning their teeth after eating sugary food or drink from their parents. Amongst them, 90 (35%) had all of their molars sound and only 8 (3%) had all four molars carious. From the remaining 176 (40.5%) children who did not get any advice from their parents, 16 (9%) had all FPMs sound and 19 (11%) had all four carious molars (Table 5). The difference between the two groups in their FPMs carious status was statistically significant (*P* < 0.05).

## 4. Discussion

It was interesting that 67% of the 9-year-old children had carious first molars, and the figure rose as the age increased and reached 70.5%, 82%, and 83.5% at 10, 11, and 12 years respectively. It is clear from this study that the carious process in the FPM starts as soon as they erupt and can be clinically observed within 1-2 years. A previous study done in Japan [16] during 1990 reported a 50% prevalence of caries in the FPM amongst 11- and 12-year-old children. This was considerably lower than the 81% found in this study. Possible reasons for the high prevalence could be due to changes in socioeconomic factors between the two groups, the Japanese culture which differs from the Saudi, Indian, and Pakistani culture and the diet which differs among these nations. These results emphasize the importance of early intervention and educational programmes which should be implemented even before the FPMs erupt (4- to 5-year-old children).

Noronha et al. [25] and Wyne [26] reported that 87% and 86% 12-year-old children had the permanent first molar affected by caries respectively. These results were similar to our findings which reported a prevalence of 83%. Many previous studies reported that aging is accompanied with increase of the caries prevalence of the FPMs among children [15, 25, 27]. It is however alarming to note that over 80% of children in this age group require either dental restorations or extractions. Given the cost of dental treatment, the time, and the resources required, the treatment will be extremely costly for the government and emphasizes the need and importance for preventive programmes.

The reasons for the high caries prevalence in the FPM could be due to various reasons such as the deep pits and fissures on the occlusal surface, the large-sized crown which leads to accumulation of acid produced by bacteria, and the early eruption of the tooth. An early preventive program like application of fissure sealants and the use of fluoride among primary school children could help reduce the prevalence of caries in these teeth [28]. Wyne [26] reported that, as the age of the children increased and they were exposed to cariogenic factors, more and more teeth become carious. Due to their anatomical structure, early eruption, and positioning in the mouth as well as *Streptococcus mutans* levels in the mouth, FPMs were observed to be highly susceptible to carious attack [25]. The earlier the child visits the dentist, the greater his or her chance exists of being free of caries. An early first dental visit may ensure that the dentist can perform preventative measures such as the application of fluoride and fissure sealants, provide oral hygiene instructions, and motivate the parents and their children regarding proper oral hygiene maintenance and dietary control as well as importance of regular visits to the dentist.

Among those who knew when the FPM erupted, 32.5% had sound FPMs and only 6.5% had all FPMs carious. Of the children who visited the dentist, only 12% received advice regarding cleaning their teeth after eating sugary food or drink. One possible reason could be that the dentist was very busy so he did not get time to offer advice. It could also mean that the dental staff feels that oral health education is not beneficial and hence not necessary to be administered.

A surprising result was the relatively high number (51%) of children who had visited a dentist in the last six months. Jeddah is one of the poorer communities in the Sharfia district and the dental services are costly. However, half of this population reported to have visited one recently. This could be due to many reasons; possibly some of the children answered in the positive as they were being examined by a dentist and felt that they had to answer positively. Another possible reason could be due to the high prevalence of caries in this community which resulted in pain, and hence children were “forced” to visit the dental team. Among those who visited the dentist in the last 6 months, 29.5% were found to have all FPMs sound, and the figure for those who received advice from the dentist of cleaning teeth after eating sugary food or drink was 37.5%. Many studies reported that regular visit of dentists leads to better oral health and condition of permanent teeth as compared to those who never visited the dentist [13, 15, 29]. 

More than 50% of the respondents reported that their parents advised them about cleaning their teeth, and, amongst them, 35% had sound FPM. Other studies have also confirmed that if good oral hygiene practices are initiated and maintained at home, it is more likely to result in lower caries prevalence [25]. Al-Shammery et al. [17] reported a higher prevalence of caries amongst molars in primary school children whose parents had a primary level of education or illiterate. Good dental care, such as correct brushing technique especially after eating sugary food or drink and consuming fewer snacks during the day resulted in lower decay [29]. As a result of this, it is essential to educate the parents and teachers regarding oral health. This could ensure that oral hygiene educational messages are constantly reinforced, and preventive programmes can be managed and maintained.

An early prevention program at the age of 6-7 years reduced caries prevalence in permanent molars [30–32]. Restorative and preventive regimens for teeth must be based on frequent recall examinations of not more than 6 monthly intervals to reduce dental decay and further caries progress in the FPMs among children. First permanent molars are very important teeth in the mouth for maintaining the integrity of the dental arches and therefore they need special attention during dental examination and careful preventive strategies including fissure sealant, topical fluoride applications, and meticulous home care.

## 5. Conclusion

The prevalence of caries in the FPMs was high. It increased as the age of the child increased. Children, who visited the dentist and received advice from them or their parents regarding oral hygiene, had less caries compared to those who did not.

## 6. Recommendations

Due to the generalized high cries prevalence amongst this cohort of students, the following programmes and interventions have been recommended for the Sharfia region in Jeddah.

1. The provision of dental extractions and restorations for all those who have been diagnosed with caries. This could be achieved by either a mobile or fixed dental clinic.
2. At least one oral hygienist should be employed on a full-time basis to visit the schools, screen the children, refer if necessary, and initiate appropriate oral hygiene and dental educational programmes.
3. By referring only those who require dental treatment, it would reduce the load on the dentist and he/she would be able to work more efficiently and effectively.
4. Teachers must be involved in the school brushing and educational programmes. School children are usually influenced by their teachers, and if the teachers promote good oral hygiene habits, it is likely that the children would accept and start implementing them. If the teachers are actively involved in the oral hygiene programmes, they would feel empowered and ensure that these programmes are carried out regularly and maintained.
5. The role of parents has also been highlighted, and parents should be invited regularly for presentations on oral and general health.
6. Fissure sealant programmes should be implemented on children as early as 6 years old (grade 1). This would reduce the caries prevalence of the FPM and prevent them from being extracted.
7. There should be preventive programmes implemented at crèche level so that the children start improving their oral hygiene habits and knowledge which could prevent dental caries later on in their lives.
8. The school headmaster and staff members should be informed about the dangers of sugary foods and their effects on oral and general health. Together, with the dental team, policies regarding the contents of the children's lunch boxes and the sale of these items in the school canteens should be initiated and implemented.

## Figures and Tables

**Table 1:** Number (%) of children who presented with and without caries of the first permanent molar in relation to age (*N* = 432).

Age in years	All molars sound		One or more carious molars		One molar carious		Two molars carious		Three molars carious		All molars carious		Total	
	*N*	%	*N*	%	*N*	%	*N*	%	*N*	%	*N*	%	*N*	%
9	36	33	72	67	28	26	28	26	14	13	2	2	108	100
10	32	30	76	70	36	34	26	24	8	7	6	5	108	100
11	20	19	88	81	24	22	36	34	20	19	8	6	108	100
12	18	17	90	83	24	23	30	28	25	23	11	9	108	100

Total	106	25	326	75	112	26	120	28	67	15	27	6	432	100

Chi-square (*χ* ^2^) = 30.3, *P* < 0.05 significant.

**Table 2:** Caries status of first permanent molars in relation to knowledge of eruption time of the first permanent molars (*N* = 432).

Do you know eruption of first permanent molars?		All molars sound		One molar carious		Two molars carious		Three molars carious		All molars carious		Total	
		*N*	%	*N*	%	*N*	%	*N*	%	*N*	%	*N*	%
Yes	18.%	26	32	16	20	20	25	13	16	5	7	80	100
No	81%	80	23	96	27	100	28	54	15	22	7	352	100

Total	100%	106	25	112	26	120	27	67	15	27	7	432	100

Chi-square (*χ* ^2^) = 4.17, *P* > 0.05.

**Table 3:** Caries status of first permanent molar in relation to visiting the dentist in the last 6 months (*N* = 432).

Did you visit the dentist in the last 6 months?			All molars sound		One molar carious		Two molars carious		Three molars carious		All molars carious		Total	
	*N*	%	*N*	%	*N*	%	*N*	%	*N*	%	*N*	%	*N*	%
Yes	222	51	66	30	70	32	42	18	37	17	7	3	222	100
No	210	49	40	19	42	20	78	37	30	14	20	10	210	100

Total	432	100	106	25	112	26	120	27	67	16	27	6	432	100

Chi-square (*χ* ^2^) = 30.0, *P* < 0.05 significant.

**Table 4:** Caries status of the first permanent molar in relation to receiving advice from the dentist about cleaning their teeth after eating sugary foods or drinks (*N* = 222).

Did you receive advise from your dentist about cleaning your teeth?		All molars sound		One carious molar		Two carious molars		Three carious molars		All molars carious		Total	
		*N*	%	*N*	%	*N*	%	*N*	%	*N*	%	*N*	%
Yes	12%	10	37	8	28	5	19	2	8	2	8	27	100
No	88%	45	23	49	25	57	29	31	17	23	6	195	100

Total	100%	55	25	57	26	62	27	33	16	25	6	222	100

Chi-square (*χ* ^2^) = 8.96, *P* < 0.05 significant.

**Table 5:** Caries status of the first permanent molar in relation to receiving advice from the parents about cleaning their teeth after eating sugary foods or drinks (*N* = 432).

Did you receive advice from your parents about cleaning your teeth?		All molars sound		One carious molar		Two carious molars		Three carious molars		All molars carious		Total	
		*N*	%	*N*	%	*N*	%	*N*	%	*N*	%	*N*	%
Yes	59.5%	90	35.0	76	30.0	54	21.0	28	11.0	8	3.0	256	100
No	40.5%	16	9.0	36	20.5	66	37.5	39	22.0	19	11.0	176	100

Total	100%	106	24.5	112	26.0	120	27.5	67	15.5	27	6.5	432	100

Chi-square (*χ* ^2^) = 60.69, *P* < 0.05 significant.
